# Artificial Intelligence for Chronic Disease Prevention and Management in Sub-Saharan Africa: A World Health Organization Health Systems Building Blocks Perspective

**DOI:** 10.1016/j.mcpdig.2026.100396

**Published:** 2026-08-17

**Authors:** Hubert Amu, Joshua Oppong

**Affiliations:** Department of Population and Behavioural Sciences, Fred N. Binka School of Public Health, University of Health and Allied Sciences, Hohoe, Ghana

Sub-Saharan Africa (SSA) is experiencing a rapid epidemiological transition, with noncommunicable diseases (NCDs) such as hypertension, diabetes, cardiovascular disease, and chronic respiratory conditions, contributing to the increase of morbidity, mortality, and long-term disability across the region.^1^^,^^2^ Primary health care (PHC) faces structural limitations such as fragmented follow-up systems, constrained workforce capacity, and underutilization of routine health data for risk stratification and clinical decision making in delivering continuous and coordinated chronic disease care.^3^

Recent advances in artificial intelligence (AI) such as machine learning, predictive analytics, and automated decision-support tools have generated considerable interest in their potential to improve chronic disease prevention and management.^4^^,^^5^ However, much of the existing discourse remains focused on disease-specific or facility-based clinical applications, with limited attention to how such technologies may be integrated into broader PHC systems in low- and middle-income countries.

The World Health Organization (WHO) Health Systems Building Blocks (HSBB) framework offers a lens for situating AI into service delivery, workforce, health information, medicines, financing, and governance to outline pathways through which AI can support population-level NCD outcomes as a systems-strengthening tool. This commentary applies the HSBB framework to describe how AI-enabled functions may strengthen PHC-based chronic disease prevention and management across SSA.

### Rationale for a Health Systems Approach to AI in NCD Prevention

Although AI tools have shown diagnostic promise, their impact on SSA may be constrained if implementation occurs without attention to health system readiness. Chronic disease prevention and long-term management require coordinated service delivery, sustained patient engagement, and continuity of care across facility- and community-based platforms. These requirements are shaped by clinical tools, workforce competencies, medicine availability, functional health information systems, and governance structures.

Primary health care delivery in many SSA settings relies on nurse-led outpatient services and community health worker programs for chronic disease screening and follow-up. Routine health data collected at health facility or district levels are frequently underused for predictive planning or risk identification. Conceptualizing AI as a health system strengthening intervention rather than a stand-alone clinical aid allows for a more contextually appropriate understanding of how emerging technologies may support prevention-oriented PHC performance in SSA.

### Mapping AI Applications to WHO HSBB

Except where a specific study is cited, the country examples that follow illustrate how existing PHC structures and digital health initiatives could support each building block; they describe potential applications rather than AI systems already deployed at scale for NCD care. The Figure summarizes how each building block maps to an illustrative AI application.

**Figure:**
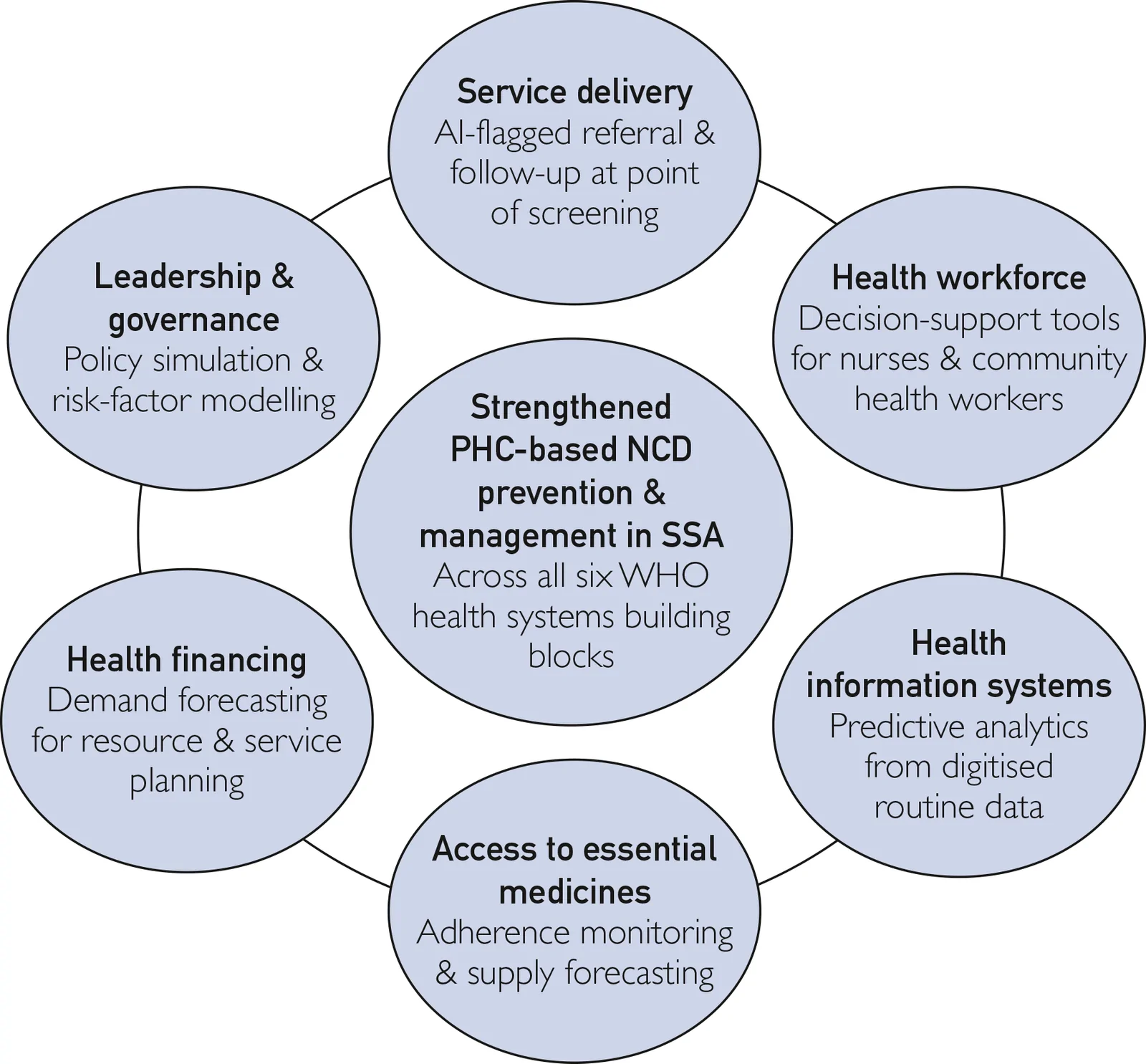
Illustrative mapping of artificial intelligence (AI) applications onto the World Health Organization (WHO) health systems building blocks for noncommunicable disease (NCD) prevention and management in sub-Saharan Africa (SSA). PHC, primary health care.

#### Service Delivery

Artificial intelligence-enabled triage systems can flag patients at elevated risk of hypertension or diabetes at the point of screening, rather than after delayed centralized review, which is how such tools shorten time to referral and follow-up. A risk-stratification model built on Ethiopian population data demonstrates this approach for hypertension.^6^ For diabetic retinopathy, an AI system grading retinal photographs achieved clinically acceptable performance in detecting referable disease in an underresourced Zambian population,^7^ and a randomized controlled trial in Tanzania is testing whether an immediate AI-generated referral decision, instead of the usual 2- to 4-week wait for manual grading, increases the share of patients who attend follow-up care.^8^ Embedding similar automated flags within existing community-based NCD screening platforms, such as those in Kenya, Ghana, and Rwanda, could shorten the screening-to-treatment interval without new infrastructure.

#### Health Workforce

Task-shifting remains a core strategy for addressing health workforce shortages in SSA.^9^ Nurse- and community health worker-led management of chronic conditions is increasingly common across countries such as Ethiopia, Uganda, and Ghana. Artificial intelligence-assisted clinical decision-support tools may provide frontline providers with real-time guidance for risk assessment, medication titration, and follow-up scheduling. In Ethiopia, health extension workers may benefit from AI-supported tools that flag individuals requiring referral for NCD assessment, while nurse-led outpatient NCD clinics in Uganda could integrate automated risk calculators to support treatment planning and monitoring. Across diverse workforce configurations, such platforms may strengthen frontline capacity to implement standardized chronic disease protocols, particularly in rural and resource-constrained settings.

#### Health Information Systems

Routine facility and district-level health data represent an underused resource for chronic disease prevention. Machine learning applied to these data may enable predictive analytics for identifying patients at risk of complications or treatment discontinuation. Achieving this is constrained by how much of SSA’s clinical record-keeping is still paper-based or partially digitized. A 10-year scoping review found infrastructure limitations and continued reliance on parallel paper registers as the dominant barriers to building the data sets that machine learning needs.^10^ Where digitization has happened at scale, it has enabled exactly this kind of use. South Africa’s Western Cape Province has linked routine outpatient, pharmacy, and laboratory data from multiple digital systems into 1 platform supporting both clinical care and in-depth analysis.^11^ Comparable linked platforms would likely need to precede similar predictive analytics elsewhere in SSA, with natural language processing helping bridge hybrid paper-digital records in the meantime.

#### Access to Essential Medicines

Medication nonadherence is a major challenge in chronic disease management across SSA. Artificial intelligence-enabled monitoring tools that identify refill gaps or irregular treatment patterns may support timely interventions to prevent treatment interruption in PHC settings. Predictive forecasting tools may additionally improve pharmaceutical supply planning at facility or district levels, which may reduce the impact of stockouts on continuity of care. These applications suggest that AI-enabled monitoring of adherence and supply continuity may strengthen treatment retention and contribute to improved disease control in settings managing hypertension and diabetes.

#### Health Financing

Artificial intelligence-based forecasting models may assist PHC systems in anticipating demand for chronic disease services and associated workforce or pharmaceutical resources. In Rwanda and Ghana, where health insurance schemes support access to NCD care, predictive modeling may inform resource allocation and service planning at district or national levels. In Nigeria and Uganda, simulation tools may support the evaluation of alternative financing arrangements for decentralized NCD care delivery. When integrated into planning processes, AI-enabled forecasting may enhance the responsiveness of PHC financing to growing chronic disease demand, enabling more efficient allocation of limited resources toward preventive care and long-term disease management.

#### Leadership and Governance

At a policy level, AI-supported modeling of population-level risk factors may inform the design and evaluation of prevention strategies targeting behavioral determinants of NCDs. Multicountry initiatives aimed at reducing population-level risk exposures may benefit from simulation tools that enable policymakers to assess potential impacts of interventions before implementation.

In South Africa and Kenya, national strategies addressing lifestyle-related risk factors may integrate predictive analytics to evaluate potential outcomes of public health interventions targeting diet or physical inactivity. In Ghana and Rwanda, policy simulation models may assist in identifying priority geographic areas for the implementation of community-based NCD prevention programs. Similarly, in Mozambique and Tanzania, modeling tools may support the evaluation of policy options aimed at reducing exposure to behavioral risk factors such as tobacco use or sedentary lifestyles. Through strengthening policy planning and evaluation processes, AI-enabled modeling tools may support the development of population-level prevention strategies tailored to local risk profiles, enhancing governance mechanisms aimed at addressing the behavioral determinants of chronic disease.

## Ethical and Implementation Considerations

Several implementation challenges must be considered in SSA contexts. Algorithmic bias arising from nonrepresentative training data may limit applicability across diverse populations.^12^ Variability in digital infrastructure and health information system interoperability may affect the feasibility of deployment across PHC facilities. Data governance frameworks must ensure the protection of patient privacy while enabling appropriate use of routine health data for predictive modeling. Addressing workforce competencies in the use and interpretation of AI-generated outputs will also be critical to responsible and equitable implementation. These considerations underscore the need for contextually grounded deployment frameworks that prioritize transparency, equity, and system-level integration.

### Implications for PHC System Strengthening in SSA

Integrating AI-enabled tools within PHC platforms may support earlier detection of chronic disease risk, improve treatment adherence, and strengthen continuity of care across facility- and community-based services. Artificial intelligence applications have the potential to enhance referral completion rates and reduce avoidable hospitalizations associated with poorly controlled NCDs when aligned with workforce capacity and health information systems. Implementation strategies should therefore prioritize system-level integration and workforce training to maximize population health impact and ensure that AI tools reinforce rather than bypass existing care pathways.

### Conclusion

Artificial intelligence offers opportunities to strengthen PHC systems for chronic disease prevention and management in SSA when deployed within existing service delivery and governance structures. Applying the WHO HSBB framework provides a systems-oriented perspective for integrating AI-enabled functions into PHC platforms. Future implementation efforts should focus on contextually appropriate deployment strategies that support continuity of care and population-level prevention of NCDs across the region.

## Potential Competing Interests

The authors report no competing interests.
